# Stress Distribution Prediction of Circular Hollow Section Tube in Flexible High-Neck Flange Joints Based on the Hybrid Machine Learning Model

**DOI:** 10.3390/ma16206815

**Published:** 2023-10-23

**Authors:** Kaoshan Dai, Hang Du, Yuxiao Luo, Rui Han, Ji Li

**Affiliations:** 1 Department of Civil Engineering, Sichuan University, Chengdu 610207, China; kdai@scu.edu.cn (K.D.); duhang202206@163.com (H.D.); hanrui9201@my.swjtu.edu.cn (R.H.); 2 GIPSA-Lab, Grenoble INP, CNRS, Université Grenoble Alpes, 38000 Grenoble, France; ji.li@gipsa-lab.grenoble-inp.fr

**Keywords:** flange joints, stress concentration factor, machine learning, random forest, ant colony algorithm

## Abstract

The flexible high-neck flange is connected to the circular hollow section (CHS) tube through welding, and the placement of the weld seam and corresponding stress concentration factor (SCF) are crucial determinants of the joint’s fatigue performance. In this study, three hybrid models combining ant colony optimization (ACO), a genetic algorithm (GA), and grey wolf optimization (GWO) with a random forest (RF) model were developed to predict the stress distribution on the inner and outer walls of the CHS tube under different flange parameter combinations. To achieve this, an automated finite element (FE) analysis program for flexible high-neck flange joints was initially developed based on ABAQUS 2020 software. Parameter combinations were randomly selected within a reasonable range to simulate the nonlinear mechanical behavior of the joint under uniform tension, generating a dataset comprising 5417 sets of data. The accuracy of the FE model was validated through experimental data from the literature. Based on this, feature importance analysis was conducted to reveal the influence of different variable parameters on the stress distribution in the tube of the joint. The flange parameters and tube stress distribution are considered as inputs and outputs, respectively. Three hybrid RF models, specifically ant colony optimization-based random forest (ACO-RF), genetic algorithm-based random forest (GA-RF), and grey wolf optimization-based random forest (GWO-RF), are trained for regression prediction. The results demonstrate that the three hybrid models outperform the original machine learning model in predictive accuracy. The ACO-RF model achieved the highest accuracy with average coefficients of determination ($R_{mean}^{2}$) of 0.9983 and 0.9865 on the testing and training sets, respectively. Building upon this foundation, the study developed a corresponding open-source graphical user interface (GUI) as a tool for facilitating computations and visualizing results. Finally, a case study on fatigue damage assessment of a flexible high-neck flange joint in a wind-turbine tower is presented to demonstrate the application of the proposed model in this study.

## 1. Introduction

### 1.1. Research Background

Flange connections represent a prevalent form of node connection in structural engineering [1]. This involves the welding of flange plates to two tubular components, subsequently joined through the use of bolts. The applicability of flange connections spans a wide range, encompassing structures as modest as streetlight poles to those as substantial as wind-turbine towers. Flange nodes can be categorized into various types based on their constructional nuances. Among these, a flexible flange pertains to a constructional w configuration wherein vertical stiffeners are not affixed to the flange plate. This results in an aesthetically pleasing, streamlined profile with reduced welding efforts and a heightened flange planarity. It is worth noting, however, that flexible flanges possess a relatively lower degree of rigidity when compared to their rigid counterparts. For structures enduring fatigue loads, the utilization of flexible flanges is a common practice to circumvent challenges stemming from fatigue initiation imperfections and residual stresses attributed to a greater number of weld seams.

Flexible flanges are typically forged in a single operation, forming both the flange plate and the flange neck, also known as flexible high-neck flanges. Under tensile loads, their characteristic of low stiffness leads to significant nonlinear mechanical behavior. This manifests as the flange plate separating under high tensile stress, generating a prying force, and inducing bending stress in the flange neck and connected tube. The widespread application of flexible high-neck flange connections is dictated by the unique requirements of different engineering projects. These distinctive features result in a wide variety of flange shapes. For instance, flange plates used in wind-turbine towers are often thick, with a substantial distance between the bolt holes and the free edge of the flange plate. These unique characteristics also give rise to variations in the nonlinear mechanical behavior of the flanges. Consequently, flange joints designed to suit specific engineering characteristics may no longer adhere to the basic assumptions of universally applicable flange engineering design methods. Therefore, instances of flange weld seam failures have been documented (such as the wind-turbine tower accident https://www.sohu.com/a/311598972_652081, accessed on 20 September 2023).

**Figure 1:**
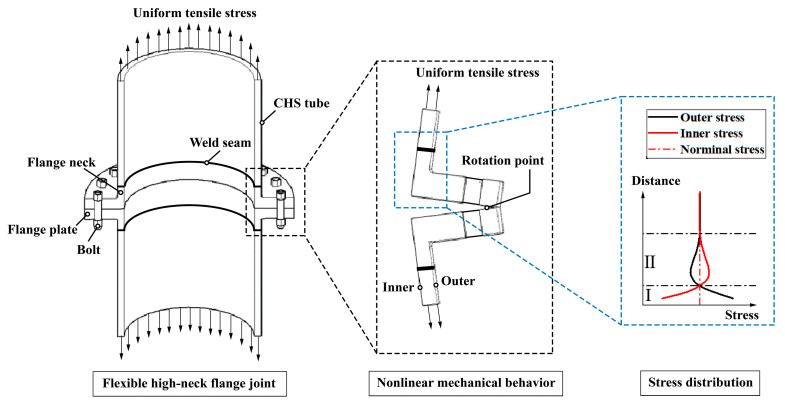
The stress distribution in the tube wall for flexible high-neck flange joint.

The forging dimensions of flexible high-neck flanges can be customized according to specific requirements. However, typically, the height of the neck is kept relatively short to save costs. In fact, for structures facing significant fatigue issues, the height of neck determines the location of the weld seam between the flange and the tube, along with the corresponding stress concentration factor (SCF). This aspect has not yet received adequate attention in current structural design [2]. Some designs even overlook the asymmetry of the flange’s inner and outer shape, as well as the stress concentration resulting from nonlinear mechanical behavior (i.e., bending stress). Instead, they simply assume that the SCF at this weld seam position is solely influenced by external parameters like the weld bead height and weld toe radius. Therefore, conducting a detailed study of the stress distribution in the tube wall (as shown in Figure 1) for flexible high-neck flange joints under different parameter combinations is essential for devising joint design solutions that prioritize both safety and cost-effectiveness.

### 1.2. State-of-the-Art Review

#### 1.2.1. The Design and Calculation Methodology for Flexible Flange Joints

Under high tensile stress, flexible flange joints exhibit nonlinear mechanical behavior, thus demanding special attention in the calculation and design process. When the tensile loading exceeds the sum of the compressive forces generated by bolt pretension, two mutually compressed flange plates will separate and open. As the tension continues to increase, the flange plates will rotate around a specific axis, and this rotation axis is different for the inner and outer flanges. Typically, the specific position of the rotation axis usually needs to be determined based on finite element analysis. Relevant design standards, such as GB50135-2017 [3], account for the nonlinear effects of this type of connection. Through the comparison of various typical flexible flange calculations, the rotation axis for the outer flange compression zone is defined at the tangent line inside the tube wall, while the rotation axis for the inner flange is defined at a position two-thirds of the tube radius away from the center. The aforementioned calculations have guided the engineering design of numerous flexible flange nodes. However, in reality, as the application scenarios of flexible flange nodes become increasingly diverse, flanges under existing parameter combinations may have already exceeded the “typical” definitions established during standard formulation. This is especially true for wind-turbine towers with thick flange plates and thin tower cylinder walls, for which further targeted research is needed on the position of the rotation axis and the nonlinear mechanical behavior of the joints.

Furthermore, for flexible flange joints subjected to fatigue loads, targeted efforts should be made to conduct fatigue-resistant design for weld seams and bolts. Standards and specifications such as IIW [4] and DNVGL-RP-C203 [5] define fatigue classes (FAT) for joints similar to flange connections, but they are tailored for cases where the weld seam is located at the root of the flange plate. The difference in the height of the neck in high-neck flanges, i.e., the variation in weld seam position is not considered. Additionally, due to the nonlinear mechanical behavior of flange joints under tensile stress, the corresponding FAT should be strongly correlated with the load mean value of the load cycles endured by the joint. This is an aspect that current engineering algorithms in standards have yet to address.

In summary, existing design standards are gradually becoming inadequate for the refined design of current flexible high-neck flanges. Especially for joint configurations that deviate from traditional calculation assumptions, determining rational assumptions for computational models and selecting appropriate flange design parameters to achieve an optimal design solution is an urgent issue that needs to be addressed.

#### 1.2.2. Application of Machine Learning Models in Structural Design Area

For addressing specialized structural designs that conventional standards may not cover, researchers typically propose corresponding theoretical calculation formulas through mechanism analysis and theoretical derivation. Alternatively, numerical simulations may be utilized to generate a large dataset of computational results, which can be used to fit semi-empirical or empirical formulas. For example, in the case of predicting SCF under different weld seam shapes, Luo et al. [6,7] and Wang et al. [8,9] calculated SCFs for various combinations of shape parameters and misalignments. And parameter fitting methods are then employed to obtain empirical formulas for the SCFs for butt welded joints, cruciform joints, T-shaped joints, and so on. This approach constitutes the origin of various empirical formulas for stress intensity factors, commonly used in the field of fracture mechanics. The same approach can also be applied to the study of flexible high-neck flange joints in this paper. However, this traditional method of parameter fitting usually requires initially defining the basic form of a formula. Subsequently, the values of various parameters in the formula are computed through fitting. The basic form of this formula relies on the researcher’s experience, or it may directly involve the use of high-order functions.

The era of data science has brought about a transformation in the development of various disciplines. The increase in computing capability means that previously complex problems no longer require simplified solutions. For the issue of stress distribution in flexible high-neck flange joints under tensile stress, which is influenced by numerous parameters, establishing nonlinear relationships among multiple parameters is currently one of the relatively mature techniques in the field of machine learning, especially when there exists a large dataset. As a crucial component of artificial intelligence, machine learning offers valuable methods for identifying relevant data features [10]. In recent years, the use of machine learning for regression prediction involving multiple parameters has become increasingly common, as demonstrated in references [10,11,12,13,14]. Additionally, Table 1 also illustrates the use of machine learning models in various application scenarios. Some machine learning models have shown a remarkable performance in the field of engineering. For example, they have been used to identify the degree of bolt looseness based on the sound generated when tapping the bolts [15], or for rapidly assessing the seismic damage status of steel frames [16]. Additionally, in the realm of stress distribution prediction, researchers have also employed models such as convolutional neural networks to create stress distribution prediction models tailored to different objects, e.g., reference [17,18,19], and notable results have been achieved, thereby circumventing the need for complex numerical computation processes.

It is evident that applying machine learning methods to predict the stress distribution in the CHS tube of flexible high-neck flange joints is technically feasible. Based on an extensive literature review, it is known that among various machine learning models for regression prediction, the random forest model has shown the best performance in multiple cases, as seen in references [14,20,21]. However, for the random forest model, certain initial parameters need to be defined before use, and the values of these parameters largely determine the final performance of the model. Currently, these parameters are often selected and manually adjusted based on experience, resulting in a low efficiency and the inability to achieve a globally optimal solution. To address this limitation, researchers have proposed hybrid models that combine traditional optimization algorithms with machine learning models. This approach involves using traditional optimization algorithms to select the initial parameters of the machine learning model, thereby improving the accuracy and modeling efficiency of the model. For the random forest model, some researchers have already proposed hybrid models that incorporate genetic algorithms [22] and grey wolf algorithms [25], all of which have achieved satisfactory results. Although there is currently no specific predictive model for the mechanical behavior of flexible flange joints, the existing achievements in the field of machine learning mentioned above provide crucial references for predicting the stress distribution in the CHS tube of high-neck flexible flange joints in this paper.

### 1.3. Existing Research Gaps

At present, there are few targeted and detailed studies on the influence of different geometric parameters and load levels on the nonlinear mechanical behavior of flange joints. In addition, the research on the nonlinear mechanical behavior of flange joints focuses mostly on bolts, with few studies focusing on the stress distribution on CHS tubes connected to flanges. However, with the increasing use of high-strength steel for lightweight design in engineering applications, the thickness of the CHS tube wall is also on a downward trend, and the stress distribution near the weld seam on tube wall should also be taken into account seriously. In fact, closer to the flange plate, there are higher stress peaks on the tube. This can be attributed to the geometric asymmetry of the flange and the nonlinear mechanical behavior, resulting in a stress concentration effect. This combines with the SCF caused by the weld seam shape to form the total SCF at the weld seam location of the flange joint. For joints subjected to severe fatigue loads, determining the stress distribution on the tube is fundamental for placing weld seams and accurately assessing stress amplitudes at the weld seam location. It necessitates targeted research across a wide range of flange parameters, covering commonly used shapes of flexible flange joints. It will ultimately lead to the development of a stress distribution prediction tool that can be utilized by researchers and engineers. For applications that require user interaction and usage, a graphical user interface (GUI) is especially important. The GUI achieves a more user-friendly and intuitive stress distribution prediction tool by providing graphical elements such as icons, buttons, windows, and menus.

### 1.4. Contribution of the Work

In response to the research gaps mentioned above, this paper conducts a detailed study on the nonlinear mechanical behavior of flexible high-neck flange joints under uniform tensile load, as well as the stress distribution law on the tube wall. This paper firstly compiles a Python program to control ABAQUS 2020 software to perform automated modeling analysis and stress extraction for flexible high-neck flange joints, establishes a dataset consisting of 5417 data, and reveals the typical stress distribution of the tube wall and the influence of different geometric parameters and loads on the stress distribution. On this basis, three hybrid models, namely the ACO-RF model, GA-RF model, and GWO-RF model, were established and trained to predict the stress distribution on the tube wall of a flexible high-neck flange joint. The best performing model was selected to develop a GUI tool for engineers and researchers to predict the stress distribution in the tube wall of flexible high-neck flange joints. Finally, a case study on the fatigue damage assessment of a flexible high-neck flange joint in a wind-turbine tower is presented to demonstrate the application of the proposed model in this study.

## 2. Theoretical Overview

In this chapter, an overview of the random forest, ACO-based RF model, GA-based RF model, and GWO-based RF model is provided.

### 2.1. Random Forest Model and Parameters

**Figure 2:**
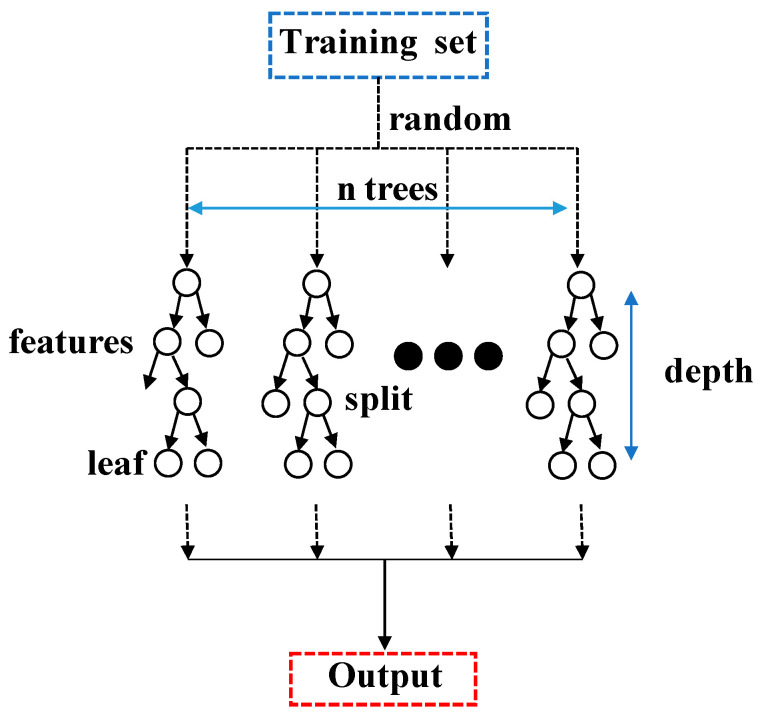
Prediction of RF model.

Random forest, introduced by Leo Breiman [26], draws its inspiration from the early work of Amit and Geman [27]. The core idea of RF is based on ensemble learning, where multiple decision trees are combined to enhance model performance. In this approach, each decision tree is constructed using a random subset of the training dataset. By averaging the regression results of each tree, the final prediction of RF is obtained, as illustrated in Figure 2.

In the RF model, there are numerous parameters that need to be determined before training, which are known as hyperparameters. These hyperparameters include: (1) The RF model allows individual decision trees to use a maximum number of features (max_features). Increasing max_features generally enhances model performance but may reduce algorithm speed. (2) A higher number of decision trees (n_estimators) in the RF model can increase model stability but also raises the overall computational load. (3) The number of leaf nodes at the end of decision trees in the RF model (min_samples_leaf) must be considered. Smaller leaf nodes make it easier for the model to capture noise in the data. (4) The maximum depth of the decision tree in the random forest (max_depth) can limit the complexity and computation of the model. (5) The minimum number of samples needed to repartition the nodes inside the decision tree in a random forest must be considered, which limits the conditions for further partitioning of the subtree. The selection of these hyperparameters is crucial for building an RF model with the best performance and generalization ability, and therefore needs to be carefully adjusted. Different combinations of hyperparameters may apply to different problems and datasets, so trade-offs need to be made in model selection and tuning.

### 2.2. Hybrid RF Model

#### 2.2.1. ACO-Base RF Model (ACO-RF)

Using ant colony optimization (ACO) for the selection of the hyperparameter of an RF model can yield stable and highly accurate regression results. The ACO algorithm was initially introduced by Marco Dorigo and his colleagues [28,29,30] in the early 1990s and is inspired by the observed behavior of ants. This algorithm mimics the cooperative behavior of ants in finding the shortest path between their nest and food sources to solve optimization problems.

**Figure 3:**
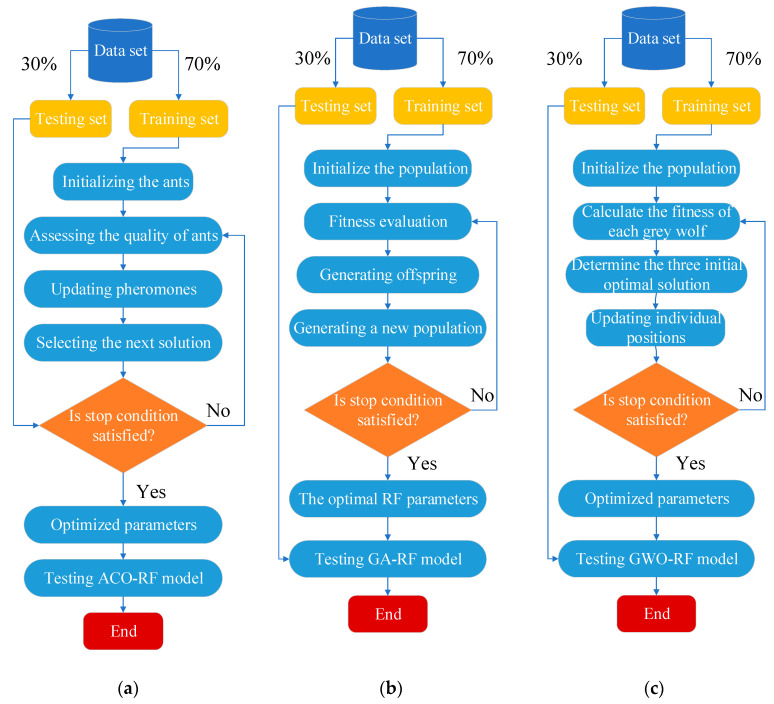
Flowchart of the three hybrid models. (**a**) ACO-RF model; (**b**) GA-RF model; (**c**) GWO-RF model.

The ACO-RF hybrid model combines the advantages of both the RF model and the ACO algorithm to optimize the hyperparameters of the RF model, leading to the optimal parameter selection results. The framework of ACO-RF, as shown in the Figure 3a, employs the ACO algorithm to search for the optimal combination of five parameters (‘max_features’, ‘n_estimators’, ‘min_samples_leaf’, ‘max_depth’, and ‘min_samples_split’) in the RF model corresponding to the dataset.

#### 2.2.2. GA-Based RF Model (GA-RF)

In addition to the ACO algorithm, this study also employed another optimization algorithm, the genetic algorithm (GA), to optimize the RF model. 

GA is a programming technique designed to create optimal solutions for any specific problem [31]. These solutions are generated by simulating the natural biological evolution process through the creation of a set of chain-like sequences resembling chromosomes. These chain-like sequences are referred to as individuals, allowing independent evaluation of each solution, followed by some random genetic operations to produce the optimal solution [32]. 

The second hybrid model and the third hybrid model used in this study, GA-RF [22], share a similar structural framework with the previous ACO-RF hybrid model. The framework of the proposed GA-RF is depicted in the Figure 3b, where GA is employed to search for the optimal combination of five parameters in the RF model corresponding to the dataset.

#### 2.2.3. GWO-Based RF Model (GWO-RF)

In addition to the ACO algorithm and GA algorithm, this study also employed another optimization algorithm, grey wolf optimization (GWO), to optimize the RF model.

The GWO algorithm [33] simulates the leadership hierarchy and hunting strategies of grey wolves in nature. This algorithm divides the wolves in the population into different ranks, to simulate a leadership hierarchy. This hierarchy is achieved through a hunting process, consisting of three main steps: searching for prey, surrounding the prey, and attacking the prey. In this simulation, each grey wolf is considered a candidate solution, and they collaborate to complete the hunting task. Among them, the grey wolf that successfully completes the hunting task is regarded as the optimal solution, representing the best individual in the current population. This simulation is inspired by the hunting behavior of grey wolf packs in the natural world. By simulating this process, the GWO algorithm can search for the optimal solutions in the search space.

Therefore, the third hybrid model used in this study, GWO-RF [26], shares a similar structural framework with the previous ACO-RF and GA-RF hybrid model. The framework of the proposed GWO-RF is depicted in the Figure 3c, where GWO is employed to search for the optimal combination of five parameters in the RF model corresponding to the dataset.

## 3. Data Generation

### 3.1. Parameter Ranges for the Geometric Model of Flange Joints

**Figure 4:**
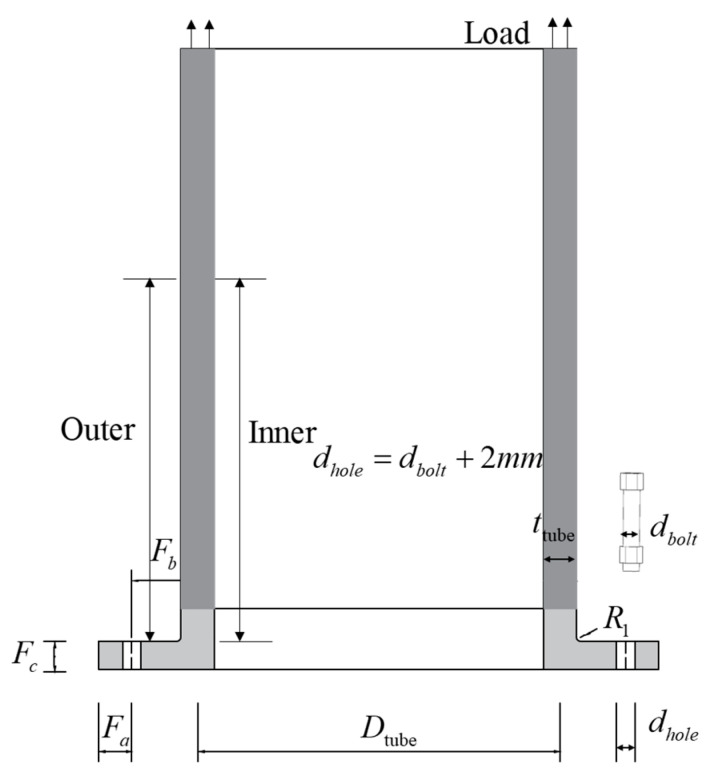
Flange geometric parameter.

To create a database for developing an RF model predicting the stress distribution of CHS tubes in flexible high-neck flange joints, a wide range of parameters has been considered, encompassing several key geometric parameters of the flange joints, as shown in Figure 4 and Table 2. It is worth noting that, in fact, these flange parameters are not entirely independent of each other. Due to the constraints imposed by flange shape, conventional design experience, and regulatory provisions on parameters, certain correlations and dependencies exist among these parameters. Consequently, flange parameters must adhere to the following constraints (see Equations (1)–(6)) for acceptance. Among these, Equation (1) limits the common diameter-to-thickness ratio of the steel pipe, Equation (2) accounts for the discrepancy between actual and standard bolt preloads, Equations (3)–(5) specify reasonable ranges for flange plate thickness and bolt hole positioning dimensions, and Equation (6) sets an upper limit on the number of bolts based on the minimum operational space required for applying bolt preload, where $P_{e}$ represents the standard preloading force of the bolt. Specific values of $P_{e}$ can be referenced in standards such as GB50017-2017 [34] and VDI2230 [35].

(1) $$8\cdot t_{tube}\leq D_{tube}\leq 66\cdot t_{tube}$$

(2) $$0.7\cdot P_{e}\leq Pretension\leq 1.3\cdot P_{e}$$

(3) $$d_{bolt}\leq F_{c}\leq 3\cdot d_{bolt}$$

(4) $$d_{bolt}\leq F_{b}\leq 3\cdot d_{bolt}$$

(5) $$d_{bolt}\leq F_{a}\leq 3\cdot d_{bolt}$$

(6) $$4\leq n_{bolt}\leq \pi \cdot (D_{tube}+2\cdot F_{b})/\left(2\cdot d_{bolt}\right)$$

**Figure 5:**
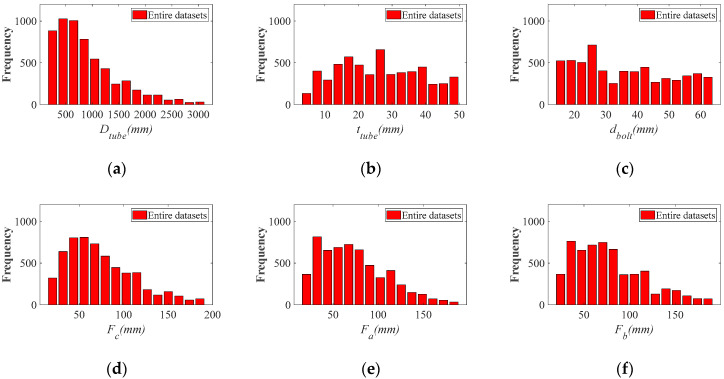

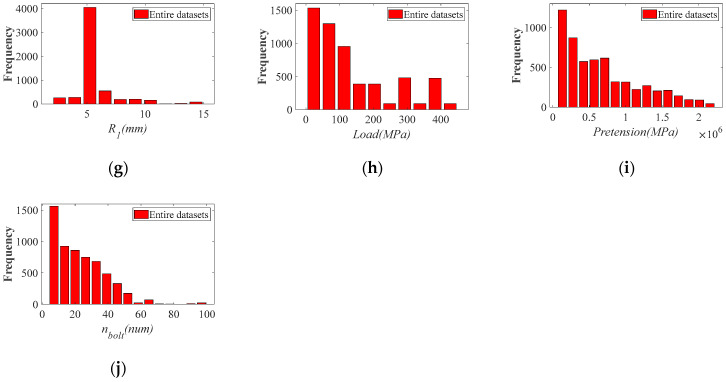
Frequency distribution of flange geometric parameters. (**a**) Distribution of variable $D_{tube}$; (**b**) Distribution of variable $t_{tube}$; (**c**) Distribution of variable $d_{bolt}$; (**d**) Distribution of variable $F_{c}$; (**e**) Distribution of variable $F_{a}$; (**f**) Distribution of variable $F_{b}$; (**g**) Distribution of variable $R_{1}$; (**h**) Distribution of variable Load; (**i**) Distribution of variable Pretension; (**j**) Distribution of variable $n_{bolt}$.

Figure 5 displays the frequency distribution of each parameter under the constraints of the aforementioned Equations (1)–(6). It is important to note that, for the fundamental parameters such as the tube diameter $t_{tube}$ and bolt diameter $d_{bolt}$, which are determined by designers based on requirements, they follow a uniform distribution. However, other parameters like $F_{a}$, $F_{b}$, $F_{c}$, *Pretension*, and $D_{tube}$ are obtained by multiplying uniform distributions of fundamental parameters with uniform distributions of multiplier coefficients, as evident from the aforementioned constraints (i.e., Equations (1)–(5)). This results in a deviation from a uniform distribution, as shown in Figure 5. As for the non-uniform distribution of parameters $R_{1}$ and *Load*, it arises from the amalgamation of databases with different parameter ranges. However, it is crucial to emphasize that, upon examination, these non-uniformities will not affect the accuracy of the subsequently trained ML model across the entire range. Under the distribution depicted in Figure 5, each parameter was randomly selected, resulting in a total of 5417 sets of parameter combinations. These combinations were then used for subsequent computational analysis and formed the database for training the hybrid ML model.

### 3.2. Numerical Analysis and Data Processing

**Figure 6:**
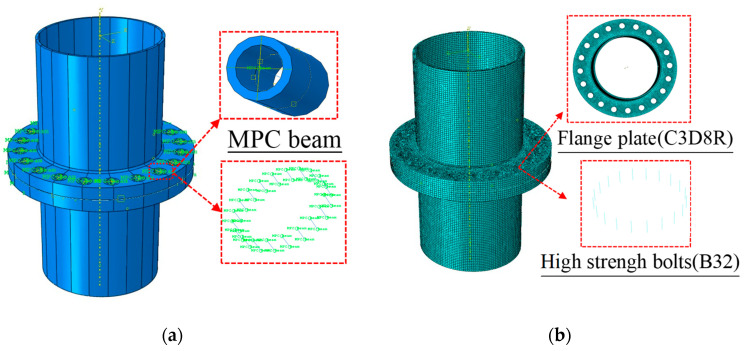
Numerical modeling and meshing strategy. (**a**) Component partitioning and contact interaction definition. (**b**) Meshing strategies.

In this study, ABAQUS 2020 software was utilized to construct the database for machine learning. A Python compiler was employed to invoke ABAQUS for modeling, analyzing, and extracting the stress results of flange joints under various combinations of parameters, as shown in Figure 6. The combinations of flange parameters were randomly selected within the ranges specified in Table 2 by the program. In the numerical model, linear elastic elements were used to simulate the flange joints. Bolts were simulated using beam elements, and multi-point constraints (MPC) were employed to establish the coupling between beam elements and multiple nodes, achieving the coupling between bolts and flanges’ plates. A second-order reduced integration element (ABAQUS name: C3D8R) was used for the flanges, and a two-node linear beam element (ABAQUS name: B31) was used for the bolts, and the contact relationship between the flange plates was established with normal hard contact, and the tangential friction coefficient was defined as 0.3.

**Figure 7:**
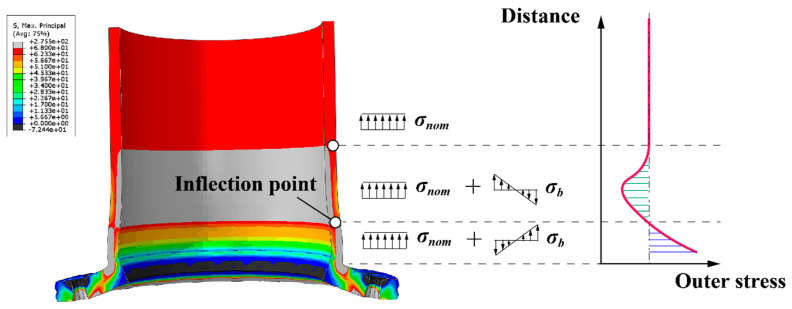
Schematic of stress components in a flange joint tube wall under uniform tensile stress.

**Figure 8:**
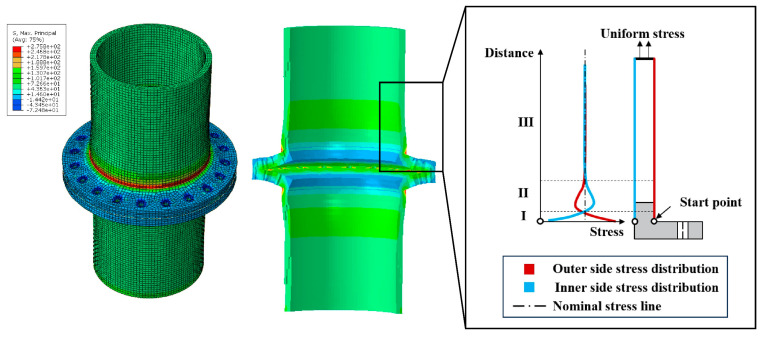
Typical FE results and stress distribution on a CHS tube.

**Figure 9:**
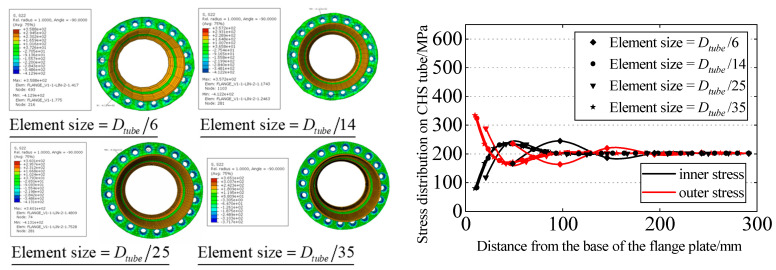
Mesh convergence analysis.

**Figure 10:**
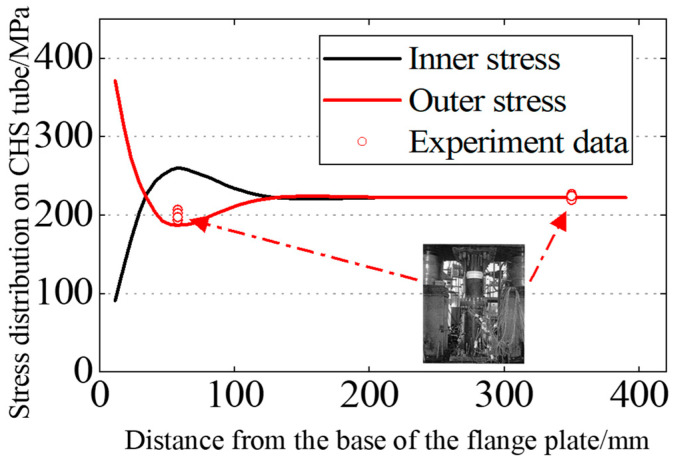
Comparison of experimental and finite element results.

Figure 7 depicts the typical stress distribution on the outer side of the tube wall, and the situation on the inner side of the tube wall is analogous. As seen from the figure, under tensile stress, near the root of the flange, the separation of the two flange plates results in bending tensile stress on the outer side of the tube wall and bending compressive stress on the inner side. However, under the loading and boundary conditions, there is an inflection point on the tube wall at a certain distance from the flange root. This leads to the appearance of bending stresses on the other side of the inflection point, which are in the opposite direction to those near the flange region. Finally, in the non-influenced area of the flange joint, the stress in the tube wall returns to the nominal stress, which is the uniform tensile stress applied to the pipe wall. The stress distribution (in terms of the maximum principal stress) of the CHS tube is extracted, starting from the intersection point of the flange plate and the neck of the high-neck flange, and the total length of extraction is chosen to be one times the diameter of tube, as shown in Figure 8. Convergence of the finite element model is achieved by adjusting the global mesh size. It can be observed that as the mesh becomes denser, the stress distribution on the inner and outer walls of the flange tube eventually converges to the same curve, as shown in Figure 9. When the mesh size reaches *D_tube_*/25, the stress calculation results have converged. Therefore, for subsequent parameter analysis, *D_tube_*/25 is chosen as the mesh size. In addition, the computed results obtained by this modeling method have been compared with the experimental data from reference [36], as shown in Figure 10. It can be seen that the simulation method in this paper has been validated by the experimental data.

## 4. Developing Machine Learning Models

### 4.1. The Performance of the Three Hybrid Models

The performance of the RF hybrid model largely depends on the selected database input variables. In this study, a total of 10 input variables were chosen based on flange dimensions ($t_{tube}$, $D_{tube}$, $F_{c}$, $F_{a}$, $F_{b}$, $R_{1}$), bolt parameters ($d_{bolt}$, $n_{bolt}$, *Pretension*), and external load size (*Load*). The output variables represent the stress distribution on the CHS tube wall of a flange joint under different geometric and load parameters, as shown in Figure 8 and Figure 9. The first principal stress at 25 nodes on the inner and outer walls is output as the output variables, totaling 50 stress points for output. To compare the performance improvement of the RF model with ACO, GA, and GWO optimization techniques, the unoptimized RF model was included in the comparison. As shown in Table 3, the values of the RF hyperparameters before and after optimization are provided. It can be observed that different optimization algorithms exhibit significant differences in optimal hyperparameters.

The open-source Python package Scikit-Learn 1.0.2 [37] was utilized to develop RF models, as well as the other three hybrid models. The entire dataset was divided into training and testing sets. A total of 3791 data points (70% of the entire dataset) were used for building predictive models, while 1626 data points (30% of the entire dataset) were employed to evaluate the predictive model performance. It is worth noting that the training and testing datasets were generated randomly using the “train_test_split” function from Scikit-Learn 1.0.2 to prevent dataset bias. To assess the performance of the four RF models mentioned above, four common metrics were selected and applied: the coefficient of determination ($R^{2}$), root mean square error (RMSE), Pearson correlation coefficient (r), and mean relative error (MRE).

**Figure 11:**
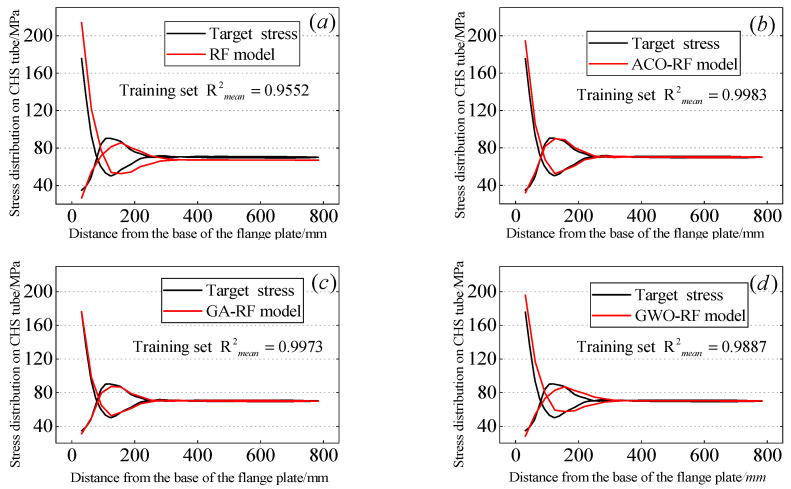
Predicted results for the training dataset: (**a**) RF, (**b**) ACO-RF, (**c**) GA-RF, (**d**) GWO-RF.

**Figure 12:**
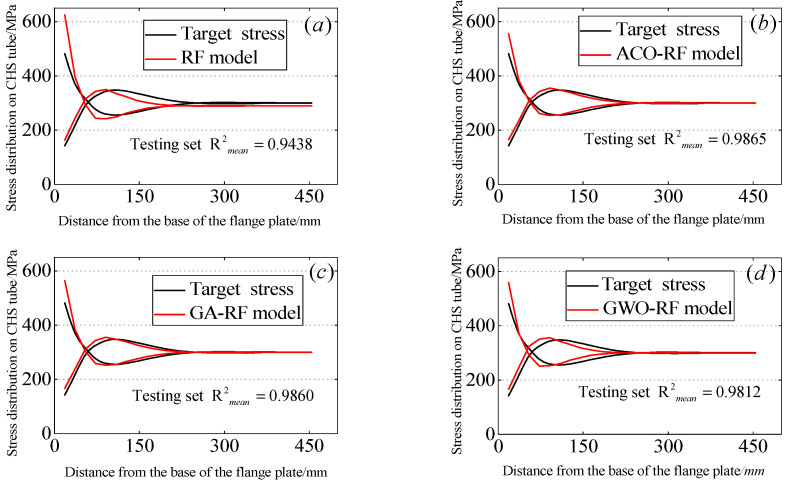
Predicted results for the testing dataset: (**a**) RF, (**b**) ACO-RF, (**c**) GA-RF, (**d**) GWO-RF.

**Figure 13:**
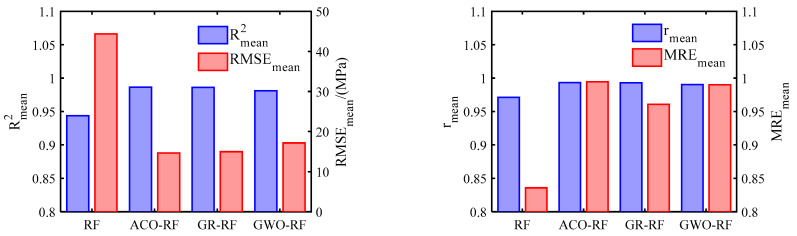
Prediction accuracy on the testing dataset of four RF models.

In the above, $y_{i}$ and ${\hat{y}}_{i}$ are respectively the target value and the predicted value of data point i, n represents the number of data, and ${\hat{y}}_{i}$ represents the mean of the target values. Since this study involves a total of 50 output variables, the mean of these output variables is used here as an evaluation metric for the regression models. Figure 11 and Figure 12 respectively illustrate the relationship between the predicted stress distribution of the flange circular pipe wall by four RF models after random sampling and the stress distribution of the CHS tube by the ABAQUS model. Table 4 summarizes the average values of the four parameters defining the regression performance of the RF models. Furthermore, the average values of the four predictive accuracy parameters of the RF models on the test dataset are presented in Figure 13. The average $R^{2}{}_{mean}$ values of their RF models are also provided. On the training dataset, the $R^{2}{}_{mean}$ values for the RF model, ACO-RF model, GA-RF model, and GWO-RF model are 0.9552, 0.9983, 0.9973, and 0.9887, respectively. On the test dataset, the $R^{2}{}_{mean}$ values are 0.9438, 0.9865, 0.9860, and 0.9812, respectively. It can be observed that the ACO-RF model produces the highest $R^{2}{}_{mean}$ values. Compared to the other RF models, the $R^{2}{}_{mean}$ of the ACO-RF on the test dataset is 4.31%, 0.1%, and 0.96% higher than the RF, GA-RF, and GWO models, respectively.

Additional details can be found in Appendix A. The results indicate that in the ACO-RF model, $RMSE_{mean}$ is the lowest, $r_{mean}$ is the highest, and $MRE_{mean}$ is closest to one, signifying the highest precision. Among the remaining three models, GA-RF exhibits the highest precision.

In conclusion, it can be stated that optimization algorithms can significantly enhance the regression performance of RF models. For the dataset created in this study, the ACO algorithm has demonstrated the most outstanding performance. However, it is important to note that the performance of these three optimization algorithms may vary for different datasets. Therefore, when applying these optimization algorithms, it is essential to consider the specific dataset characteristics and the problem context to choose the most suitable optimization approach.

### 4.2. Feature Importance Analysis

**Figure 14:**
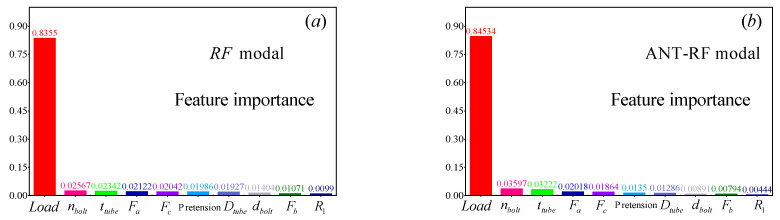

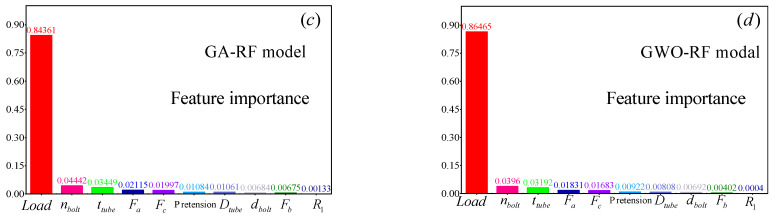
Feature importance plot obtained by the four RF models. (**a**) RF model, (**b**) ACO-RF model, (**c**) GA-RF model, (**d**) GWO-RF model.

The regression model chosen in this study is built based on the RF model, which is an efficient ensemble machine learning method capable of effectively analyzing input feature variables. The order of feature variable importance obtained from the four RF models is shown in Figure 14. The consistent order of importance, from highest to lowest, is load, $n_{bolt}$, $t_{tube}$, $F_{a}$, $F_{c}$, *Pretension*, $D_{tube}$, $d_{bolt}$, $F_{b}$, $R_{1}$. In predicting the target variable, the variable with the highest feature importance is load. In the RF model, its importance level is 0.8355; in the ACO-RF model, it is 0.84534; in the GA-RF model, it is 0.84361; and in the GWO-RF model, it is 0.86465. Such high values indicate that load plays an almost decisive role in the regression process of the model, followed by $n_{bolt}$. In the regression process of the target variable, the feature variable that has the least impact on the model is $R_{1}$, which is close to zero in all four models, indicating that this variable has minimal influence on predicting the target variable. However, it can be affirmed that although the feature importance coefficient of load is relatively high and has a decisive impact on the stress distribution values, the influence of the other parameters should not be overlooked, as they also have a decisive effect on the shape of the stress distribution.

## 5. Application of the Proposed GA-RF Model

The proposed GA-RF hybrid machine learning model can be used to predict the stress distribution of the tube wall of flexible high-neck flange joints under arbitrary geometric and load parameters, and fatigue performance evaluation is one of the important application scenarios of this model. This chapter will take the fatigue damage evaluation of a flexible high-neck flange joint in a lattice-type wind-turbine tower structure as an example to demonstrate the specific process of applying this model.

### 5.1. Engineering Background

**Figure 15:**
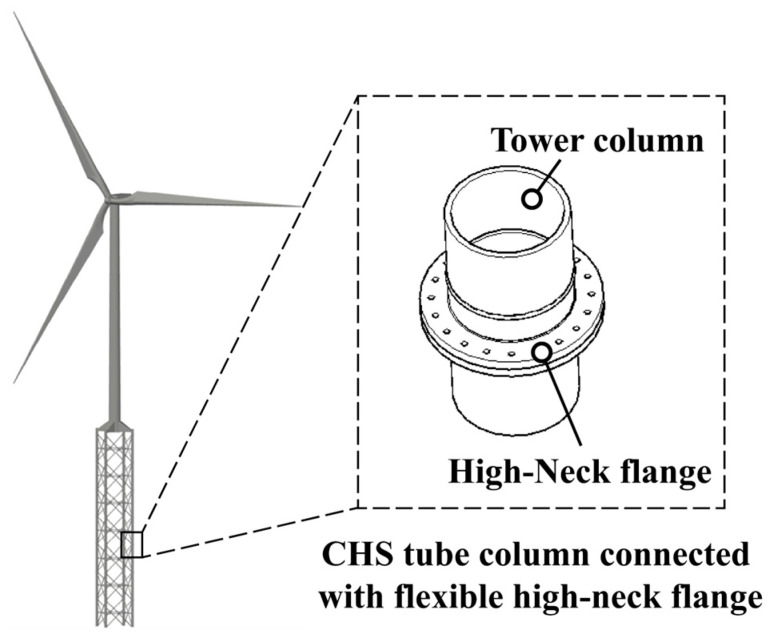
Schematic diagram of lattice-tubular hybrid wind-turbine support structure.

A large scale ultra-high wind-turbine tower with a hub height of 180 m and a rated power of 5.5 MW adopts a hybrid-type support structure scheme, with a lattice-type tower at the bottom and a tubular tower at the top. The flexible high-neck flange connection is used in the lattice part, as shown in Figure 15.

The fatigue load of the wind-turbine tower during its 20-year service life is obtained under the provisions of the IEC code [38] through wind-turbine integrated load simulation technology. The resulting fatigue load is subsequently converted into the internal forces acting on the tube members at a specific location of the flexible high-neck flange joint through structural calculations. These forces are presented in the form of a rainflow matrix, as shown in the Supplementary Materials of this paper. The rainflow matrix of fatigue load records the range, mean value, and corresponding cycle number of the axial force of the tube member that the flexible high-neck flange joint endures during its service life.

### 5.2. Fatigue Assessment Process

**Figure 16:**
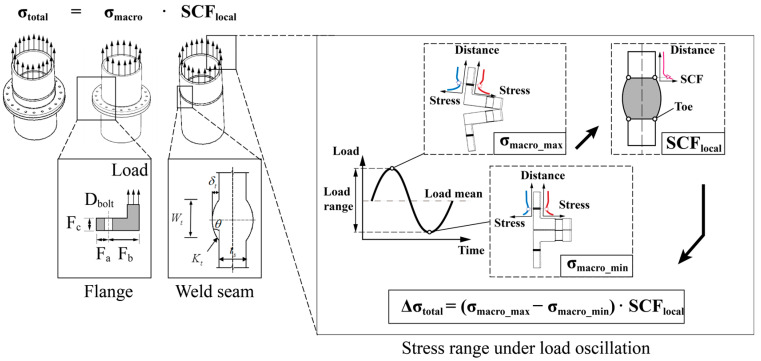
Stress composition of flexible high neck flange joint under load cycles.

Taking the “effective notch stress-fatigue life” evaluation method recommended by IIW [4] as an example, the fatigue damage of the joint during the 20-year service period is obtained by calculating the notch stress range at the weld toe of the weld seam. The stress of the flexible high-neck flange joint weld under loadings is mainly determined by two parts: the macroscopic shape of the flange joint and the nonlinearity under tensile load, which can be predicted by the hybrid machine learning model proposed in this study, and the local shape of weld seam on neck, which can be calculated using the existing SCF parametric formula, as shown in Figure 16 below.

**Figure 17:**
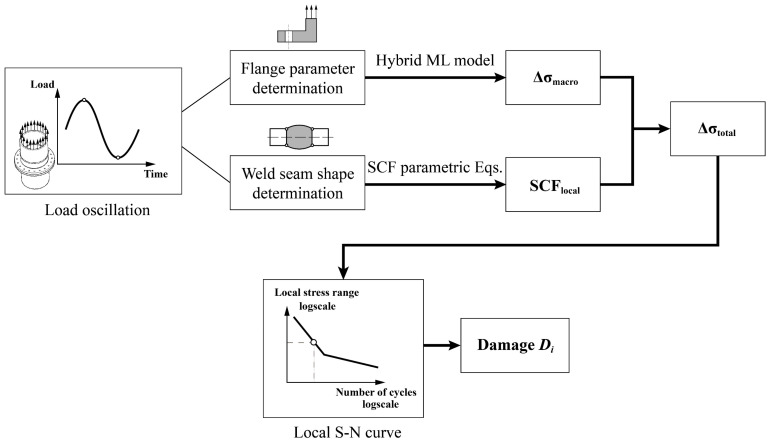
Calculation process of fatigue damage under a certain load cycle.

Therefore, for the fatigue damage calculation of the joint under a certain load cycle in this example, the following flowchart shown in Figure 17 can be used. Similarly, by repeating this process and superimposing the damage values under all load cycles based on Miner’s rule [38], the total damage value of the joint can be obtained.

### 5.3. Damage Calculation of Flange Joint

**Figure 18:**
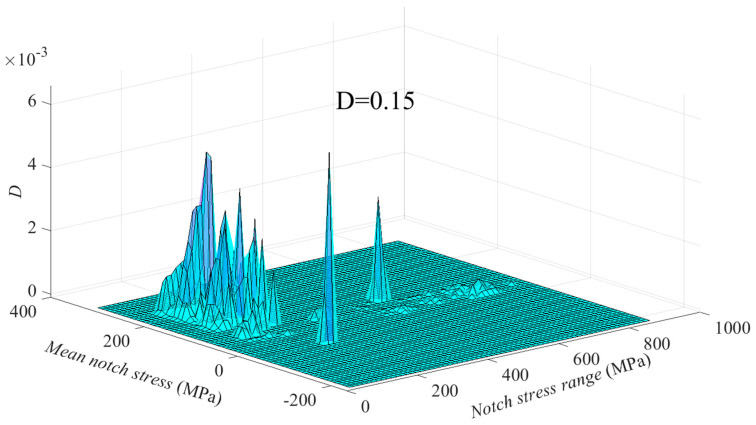
Damage rainflow matrix.

The rainflow matrix of fatigue load of a flexible high-neck flange joint of the above wind-turbine tower is shown in the Supplementary Materials of this paper. The parameters of the flange and weld seam are shown in Table 5 and Table 6, respectively. Through the GA-RF model proposed in this study and the parameter formula for SCF of butt welds proposed by the author in [6], the damage under each set of mean load and load range in the rainflow matrix can be calculated with the recommended S-N curve for the effective notch stress approach (i.e., FAT225 recommended by IIW [4]), as shown in Figure 18. Finally, the total damage is calculated by summing up the damages of all sets according to Miner’s rule [39], resulting in a value of 0.15.

## 6. Conclusions

This study developed an automated calculation program using ABAQUS 2020 software to perform stress analysis of flexible high-neck flange joints under various geometric parameter combinations. The accuracy of the finite element model was validated through experimental data collected in the literature. Subsequently, a dataset comprising over 4500 entries within reasonable parameter ranges was generated for training three hybrid models: ACO-RF, GA-RF, and GWO-RF. This enabled the prediction of stress distribution in the CHS tube of flexible high-neck flange joints.

**Figure 19:**
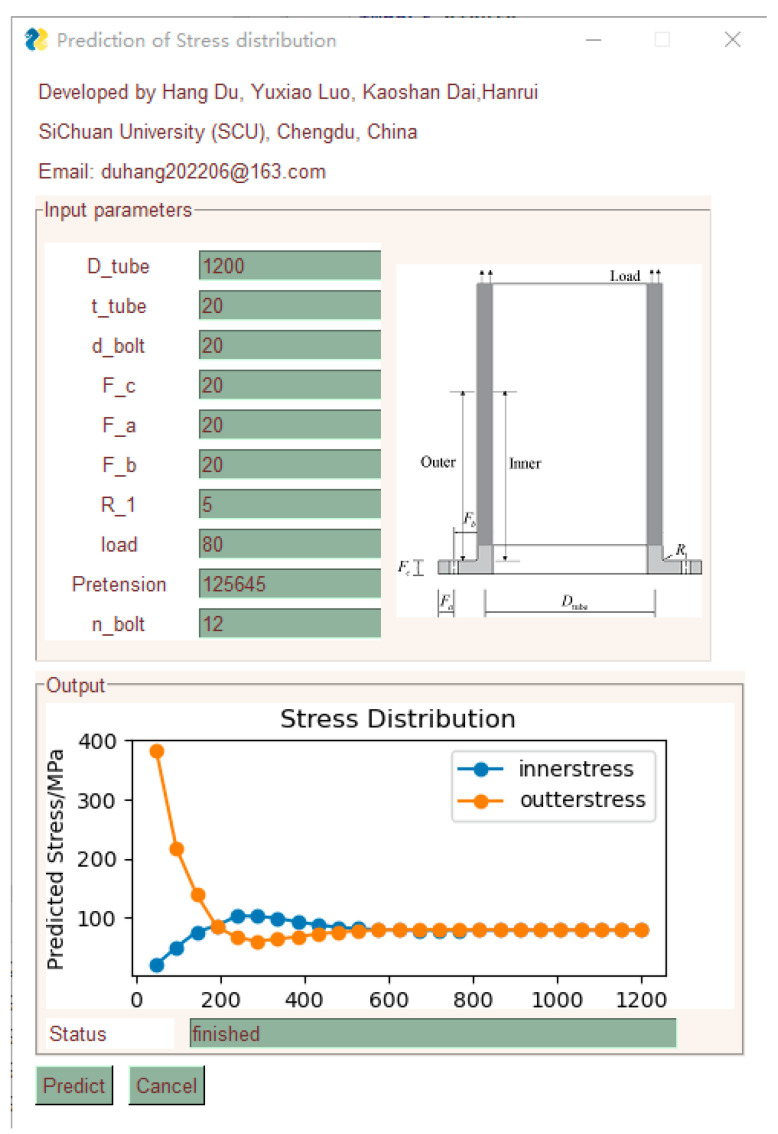
GUI for predicting the stress distribution in the CHS tube of high-neck flexible flange joints.

Through training, it was determined that the hybrid models outperform the original RF model in terms of accuracy, while also eliminating the cumbersome process of manually adjusting hyperparameters. In terms of predictive performance, the ANT-RF model demonstrated the best performance. As a result, this paper developed an open-source GUI program based on the ANT-RF model for use by engineers and researchers to predict the stress distribution in the CHS tube of flexible high-neck flange joints, as shown in Figure 19 (Download: https://github.com/Qwduang/ANT-RFmodel.git, accessed on 20 September 2023).

The following conclusions can be drawn:

1. Under tensile stress, the flexible high-neck flange exhibits significant nonlinear mechanical behavior, resulting in bending tensile stress on the outer surface of the CHS tube. Near the flange, high stress peaks can be detected. As the distance from the flange root increases, the stress level rapidly decreases to below the nominal stress, and then returns to the nominal stress level. On the inner surface of the tube, bending compressive stress is formed under tensile stress, resulting in an opposite trend compared to the outer surface.
2. Feature importance analysis reveals that the most influential parameter on the stress distribution in the tube of flange joint is the magnitude of the tensile force. In addition to this, the number of bolts, wall thickness of the tube, and the positioning dimensions of the bolt holes follow in terms of their respective levels of impact, each playing a non-negligible role in the stress distribution.
3. For the specific case discussed in this study, the performance of the ACO-RF model stands out as the most outstanding among the three hybrid models with an average coefficient of determination ($R^{2}{}_{mean}$) of 0.9865 on the test dataset.

The tool developed in this study can compute the stress distribution on the CHS tube of the flexible flange joint under different parameters and loads. It assists in determining the optimal weld seam location and SCF. It is worth mentioning that the computed results of stress distribution can be normalized into the form of an SCF, characterizing the stress concentration caused by the macro-geometry of the flange and nonlinear mechanical behavior. This SCF, in conjunction with the SCF attributed to weld seam shape and potential misalignments, collectively form the comprehensive SCF for the weld in the flexible flange joint. This comprehensive SCF assessment provides a crucial foundation for conducting fatigue evaluations, employing local stress-life approaches such as the hot-spot stress method, effective notch stress approach, and similar techniques. In the future, building upon the research presented in this paper, further exploration will be conducted on the impact of various flange parameters on bolt stresses. This will lead to the development of a comprehensive predictive model for stress distribution in flexible high-neck flange joints, encompassing both weld seams and bolts. Subsequently, this model will serve as the foundation for assessing the competitive mechanisms of fatigue failure between weld seams and bolts in this joint configuration, as well as for conducting fatigue reliability evaluations at the joint level.

## Figures and Tables

**Table 1:** Machine learning models in various application domains.

Authors	Machine Learning Model Used	Application Scenario
Mangalathu [10]	Artificial Neural Network (ANN)	Failure model prediction of a round reinforced concrete bridge column
Rahman [11]	eXtreme Gradient Boosting (XGboost)	Prediction of shear strength of steel fiber reinforced concrete beams
Kameshwar [13]	Decision Tree (DT)	Earthquake recovery model of a bridge
He [15]	K-Nearest Neighbor (KNN)	Prediction of loosening state of underwater bolt connection
Nguyen [16]	Random Forest (RF)	Damage assessment of buildings after an earthquake
Bolandi [17]	Convolutional Neural Networks (CNN)	Stress distribution of damaged structural components with stress concentration
Liang [18]	Deep Learning Model (DL)	Stress distribution in human aorta
Sepasdar [19]	Convolutional Neural Networks (CNN)	Nonlinear stress distribution and failure modes in microstructure characterization of composite materials
Nguyen [20]	Random Forest (RF)	Maximum displacement of three pendulum isolation system
Nguyen [21]	Random Forest (RF)	Peak lateral displacement of an isolation system under earthquake action
Zhou [22]	Random Forest (RF)	Assess the liquefaction potential of soil
Leleń [23]	Linear Regression (LR)	Prediction of vibration amplitude and surface roughness after water jet cutting
Ahmed [24]	Random Forest (RF)	Predict the strength of self-compacting mortar samples

**Table 2:** Ranges of flange geometric parameters.

Notation	Unit	Range of Variable	Description
$t_{tube}$	mm	6–46	Thickness of steel pipe
$D_{tube}$	mm	173–2555	Steel pipe diameter
*Pretension*	N	59,886–2,034,355	The pretension force applied by the bolt
$d_{bolt}$	mm	14–64	Diameter of bolt
$F_{c}$	mm	17.45–174.17	Flange thickness
$F_{a}$	mm	17.92–157.87	Distance from hole center to flange edge
$F_{b}$	mm	21.38–150.8	Distance from hole center to root
$n_{bolt}$	num	4–96	Number of bolts
$R_{1}$	mm	2–13	Flange fillet
*Load*	MPa	0–450	A uniform load applied to a steel pipe

**Table 3:** The optimal parameters of three hybrid models and unoptimized general parameters.

Model	n_Estimators	Max_Features	Min_Samples_Leaf	Max_Depth	Min_Samples_Split
RF	100	0.2	10	200	2
ACO-RF	110	1.0	2	91	4
GR-RF	133	0.7	1	14	3
GWO-RF	161	0.6	3	15	4

**Table 4:** Prediction accuracy of four RF models in the training and testing datasets.

Dataset	Parameters	Model			
		RF	ACO-RF	GA-RF	GWO-RF
Training	$R^{2}{}_{mean}$	0.9552	0.9983	0.9973	0.9887
	$RMSE_{mean}(MPa)$	41.4376	5.6718	6.8446	13.8133
	$r_{mean}$	0.9773	0.9991	0.9986	0.9943
	$MRE_{mean}$	0.8396	0.9929	0.9905	0.9901
Testing	$R^{2}{}_{mean}$	0.9438	0.9865	0.9860	0.9812
	$RMSE_{mean}(MPa)$	44.3442	14.6456	14.9875	17.1670
	$r_{mean}$	0.9713	0.9932	0.9929	0.9903
	$MRE_{mean}$	0.8358	0.9946	0.9608	0.9900

**Table 5:** Parameters of the flange joint.

$t_{tube}$	$D_{tube}$	*Pretension*	$d_{bolt}$	$F_{c}$	$F_{a}$	$F_{b}$	$n_{bolt}$	$R_{1}$
20 mm	977 mm	339,500 N	36 mm	50 mm	60 mm	75 mm	22	5 mm

**Table 6:** Parameters of the weld seam.

$W_{t}$	θ	$\delta_{t}$	$t_{s}$	ρ
37.5 mm	30°	6.125 mm	25 mm	1 mm

## Data Availability

Data are available upon request from the corresponding author.

## Supplementary Materials

The following supporting information can be downloaded at: https://www.mdpi.com/article/10.3390/ma16206815/s1.

## Appendix A

The performance of each of the 50 output variables for the RF model, ACO-RF model, GA-RF model, and GWO-RF model is presented in Appendix A Table A1 and Appendix A Table A2.

**Table A1:** Detailed prediction accuracy of four RF models in the training datasets.

Dataset	Output	Parameters	RF	ACO-RF	GA-RF	GWO-RF
Training	Inner stress1	$R^{2}$	0.8694	0.9877	0.9781	0.9032
		RMSE(MPa)	29.317	8.0435	10.6888	21.9770
		r	0.9324	0.9938	0.9890	0.9504
		MRE	0.7971	0.9467	0.9374	0.9369
Training	Inner stress2	$R^{2}$	0.9083	0.9930	0.9873	0.9441
		RMSE(MPa)	39.2002	9.2257	12.3288	25.5521
		r	0.9530	0.9965	0.9936	0.9716
		MRE	0.8181	0.9448	0.9575	0.9607
Training	Inner stress3	$R^{2}$	0.9384	0.9958	0.9925	0.9702
		RMSE(MPa)	42.249	8.8295	11.6865	23.1952
		r	0.9687	0.9979	0.9963	0.9850
		MRE	0.8395	0.9891	0.9867	0.9838
Training	Inner stress4	$R^{2}$	0.9492	0.9969	0.9947	0.9807
		RMSE(MPa)	45.1662	8.5487	11.1194	21.2575
		r	0.9743	0.9985	0.9974	0.9903
		MRE	0.8393	0.9933	0.9916	0.9885
Training	Inner stress5	$R^{2}$	0.9524	0.9977	0.9967	0.9868
		RMSE(MPa)	47.5791	7.8710	9.4064	18.9266
		r	0.9759	0.9989	0.9984	0.9934
		MRE	0.8379	0.9946	0.9932	0.9903
Training	Inner stress6	$R^{2}$	0.9581	0.9985	0.9977	0.9907
		RMSE(MPa)	43.6960	6.1540	7.4928	15.1826
		r	0.9788	0.9992	0.9989	0.9953
		MRE	0.8397	0.9962	0.9948	0.9925
Training	Inner stress7	$R^{2}$	0.9610	0.9988	0.9981	0.9922
		RMSE(MPa)	41.3426	5.2536	6.5508	13.3918
		r	0.9803	0.9994	0.9991	0.9961
		MRE	0.8401	0.9971	0.9956	0.9932
Training	Inner stress8	$R^{2}$	0.9622	0.9990	0.9982	0.9923
		RMSE(MPa)	38.6814	4.6124	6.0639	12.5662
		r	0.9809	0.9995	0.9991	0.9962
		MRE	0.8409	0.9974	0.9957	0.9933
Training	Inner stress9	$R^{2}$	0.9649	0.9992	0.9986	0.9940
		RMSE(MPa)	36.5458	3.9847	5.1691	10.7202
		r	0.9823	0.9996	0.9993	0.9970
		MRE	0.8417	0.9982	0.9968	0.9938
Training	Inner stress10	$R^{2}$	0.9663	0.9993	0.9988	0.9947
		RMSE(MPa)	34.9192	3.6221	4.6894	9.7043
		r	0.9830	0.9996	0.9994	0.9974
		MRE	0.8424	0.9986	0.9973	0.9943
Training	Inner stress11	$R^{2}$	0.9680	0.9994	0.9990	0.9958
		RMSE(MPa)	33.7760	3.2470	4.1320	8.4678
		r	0.9839	0.9997	0.9995	0.9979
		MRE	0.8430	0.9991	0.9980	0.9948
Training	Inner stress12	$R^{2}$	0.9691	0.9995	0.9992	0.9965
		RMSE(MPa)	32.9709	2.9536	3.7419	7.6019
		r	0.9845	0.9997	0.9996	0.9983
		MRE	0.8433	0.9993	0.9983	0.9950
Training	Inner stress13	$R^{2}$	0.9699	0.9995	0.9993	0.9971
		RMSE(MPa)	32.5265	2.7246	3.4444	6.9611
		r	0.9849	0.9998	0.9996	0.9985
		MRE	0.8435	0.9994	0.9985	0.9951
Training	Inner stress14	$R^{2}$	0.9705	0.9996	0.9994	0.9974
		RMSE(MPa)	32.210	2.5262	3.1935	6.4518
		r	0.9852	0.9998	0.9997	0.9987
		MRE	0.8436	0.9994	0.9985	0.9951
Training	Inner stress15	$R^{2}$	0.9708	0.9996	0.9994	0.9977
		RMSE(MPa)	32.0628	2.3954	3.0280	6.1406
		r	0.9853	0.9998	0.9997	0.9988
		MRE	0.8436	0.9993	0.9985	0.9951
Training	Inner stress16	$R^{2}$	0.9710	0.9997	0.9995	0.9978
		RMSE(MPa)	31.9702	2.3019	2.9121	5.9312
		r	0.9854	0.9998	0.9997	0.9989
		MRE	0.8436	0.9993	0.9985	0.9951
Training	Inner stress17	$R^{2}$	0.9711	0.9997	0.9995	0.9979
		RMSE(MPa)	31.9338	2.2476	2.8450	5.8154
		r	0.9855	0.9998	0.9997	0.9990
		MRE	0.8435	0.9992	0.9985	0.9951
Training	Inner stress18	$R^{2}$	0.9712	0.9997	0.9995	0.9979
		RMSE(MPa)	31.9240	2.2190	2.8136	5.7599
		r	0.9855	0.9998	0.9998	0.9990
		MRE	0.8435	0.9991	0.9984	0.9951
Training	Inner stress19	$R^{2}$	0.9712	0.9997	0.9995	0.9980
		RMSE(MPa)	31.9227	2.2032	2.7985	5.7287
		r	0.9855	0.9998	0.9998	0.9990
		MRE	0.8435	0.9989	0.9983	0.9951
Training	Inner stress20	$R^{2}$	0.9712	0.9997	0.9995	0.9980
		RMSE(MPa)	31.9397	2.2094	2.8096	5.7390
		r	0.9855	0.9998	0.9998	0.9990
		MRE	0.8434	0.9988	0.9983	0.9951
Training	Inner stress21	$R^{2}$	0.9712	0.9997	0.9995	0.9980
		RMSE(MPa)	31.9447	2.2096	2.8113	5.7320
		r	0.9855	0.9998	0.9998	0.9990
		MRE	0.8434	0.9988	0.9983	0.9951
Training	Inner stress22	$R^{2}$	0.9711	0.9997	0.9995	0.9979
		RMSE(MPa)	31.972	2.2493	2.8520	5.7864
		r	0.9854	0.9998	0.9997	0.9990
		MRE	0.8434	0.9987	0.9983	0.9951
Training	Inner stress23	$R^{2}$	0.9711	0.9997	0.9995	0.9979
		RMSE(MPa)	31.9700	2.2451	2.8413	5.7580
		r	0.9854	0.9998	0.9998	0.9990
		MRE	0.8434	0.9987	0.9983	0.9952
Training	Inner stress24	$R^{2}$	0.9710	0.9997	0.9995	0.9979
		RMSE(MPa)	32.006	2.3232	2.9121	5.8588
		r	0.9854	0.9998	0.9997	0.9989
		MRE	0.8435	0.9991	0.9986	0.9951
Training	Inner stress25	$R^{2}$	0.9711	0.9997	0.9995	0.9980
		RMSE(MPa)	31.9666	2.1928	2.7747	5.6582
		r	0.9854	0.9999	0.9998	0.9990
		MRE	0.8436	0.9993	0.9987	0.9951
Training	Outer stress1	$R^{2}$	0.8794	0.9915	0.9898	0.9616
		RMSE(MPa)	228.0632	54.0403	57.7710	113.0485
		r	0.9377	0.9957	0.9949	0.9806
		MRE	0.8033	0.9828	0.9812	0.9729
Training	Outer stress2	$R^{2}$	0.8963	0.9941	0.9930	0.9724
		RMSE(MPa)	123.5416	25.5785	27.5240	55.2532
		r	0.9468	0.9971	0.9965	0.9861
		MRE	0.8141	0.9879	0.9863	0.9788
Training	Outer stress3	$R^{2}$	0.8910	0.9946	0.9922	0.9686
		RMSE(MPa)	84.7124	16.5371	19.4731	39.5922
		r	0.9439	0.9973	0.9961	0.9842
		MRE	0.8224	0.9897	0.9875	0.9817
Training	Outer stress4	$R^{2}$	0.8902	0.9947	0.9913	0.9619
		RMSE(MPa)	55.7043	10.7272	13.5501	28.3224
		r	0.9435	0.9974	0.9956	0.9808
		MRE	0.8328	0.9797	0.9787	0.9803
Training	Outer stress5	$R^{2}$	0.9095	0.9955	0.9928	0.9699
		RMSE(MPa)	42.8242	8.1437	10.1963	20.7499
		r	0.9537	0.9977	0.9964	0.9848
		MRE	0.8406	0.9747	0.9674	0.9823
Training	Outer stress6	$R^{2}$	0.9331	0.9967	0.9946	0.9781
		RMSE(MPa)	35.2476	6.3658	8.0538	16.2047
		r	0.9660	0.9983	0.9973	0.9890
		MRE	0.8444	0.9765	0.9837	0.9856
Training	Outer stress7	$R^{2}$	0.9465	0.9975	0.9960	0.9836
		RMSE(MPa)	34.2433	5.7277	7.2509	14.6532
		r	0.9729	0.9988	0.9980	0.9918
		MRE	0.8444	0.9822	0.8932	0.9887
Training	Outer stress8	$R^{2}$	0.9550	0.9982	0.9971	0.9880
		RMSE(MPa)	33.5105	4.9753	6.3458	12.9147
		r	0.9772	0.9991	0.9986	0.9940
		MRE	0.8441	0.9848	0.9809	0.9892
Training	Outer stress9	$R^{2}$	0.9591	0.9986	0.9977	0.9902
		RMSE(MPa)	33.9730	4.6722	5.9011	12.1572
		r	0.9793	0.9993	0.9988	0.9951
		MRE	0.8434	0.9679	0.9892	0.9909
Training	Outer stress10	$R^{2}$	0.9631	0.9989	0.9983	0.9928
		RMSE(MPa)	33.5021	4.0937	5.1244	10.6128
		r	0.9814	0.9995	0.9992	0.9964
		MRE	0.8435	0.9906	0.9901	0.9921
Training	Outer stress11	$R^{2}$	0.9655	0.9991	0.9986	0.9942
		RMSE(MPa)	33.3807	3.7771	4.6846	9.6466
		r	0.9826	0.9996	0.9993	0.9971
		MRE	0.8434	0.9957	0.9943	0.9929
Training	Outer stress12	$R^{2}$	0.9673	0.9993	0.9989	0.9953
		RMSE(MPa)	33.0116	3.4241	4.2419	8.6729
		r	0.9835	0.9996	0.9994	0.9977
		MRE	0.8436	0.9979	0.9964	0.9935
Training	Outer stress13	$R^{2}$	0.9685	0.9994	0.9991	0.9961
		RMSE(MPa)	32.7939	3.1800	3.9217	7.9358
		r	0.9841	0.9997	0.9995	0.9981
		MRE	0.8437	0.9985	0.9971	0.9938
Training	Outer stress14	$R^{2}$	0.9693	0.9995	0.9992	0.9967
		RMSE(MPa)	32.5636	2.9670	3.6536	7.3535
		r	0.9846	0.9997	0.9996	0.9983
		MRE	0.8438	0.9990	0.9976	0.9940
Training	Outer stress15	$R^{2}$	0.9699	0.9995	0.9993	0.9970
		RMSE(MPa)	32.4160	2.8173	3.4571	6.9347
		r	0.9848	0.9998	0.9996	0.9985
		MRE	0.8439	0.9993	0.9979	0.9941
Training	Outer stress16	$R^{2}$	0.9702	0.9996	0.9993	0.9973
		RMSE(MPa)	32.2930	2.7007	3.3075	6.6378
		r	0.9850	0.9998	0.9997	0.9986
		MRE	0.8440	0.9994	0.9980	0.9941
Training	Outer stress17	$R^{2}$	0.9705	0.9996	0.9994	0.9974
		RMSE(MPa)	32.2087	2.6226	3.2109	6.4521
		r	0.9851	0.9998	0.9997	0.9987
		MRE	0.8440	0.9994	0.9981	0.9941
Training	Outer stress18	$R^{2}$	0.9706	0.9996	0.9994	0.9975
		RMSE(MPa)	32.1475	2.5650	3.1434	6.3283
		r	0.9852	0.9998	0.9997	0.9988
		MRE	0.8440	0.9994	0.9981	0.9941
Training	Outer stress19	$R^{2}$	0.9707	0.9996	0.9994	0.9976
		RMSE(MPa)	32.0967	2.5220	3.0993	6.2485
		r	0.9852	0.9998	0.9997	0.9988
		MRE	0.8440	0.9994	0.9981	0.9940
Training	Outer stress20	$R^{2}$	0.9708	0.9996	0.9994	0.9976
		RMSE(MPa)	32.0650	2.4856	3.0634	6.1844
		r	0.9853	0.9998	0.9997	0.9988
		MRE	0.8441	0.9994	0.9980	0.9940
Training	Outer stress21	$R^{2}$	0.9708	0.9996	0.9994	0.9977
		RMSE(MPa)	32.0285	2.4421	3.0235	6.1126
		r	0.9853	0.9998	0.9997	0.9988
		MRE	0.8440	0.9994	0.9980	0.9940
Training	Outer stress22	$R^{2}$	0.9709	0.9996	0.9995	0.9978
		RMSE(MPa)	32.0028	2.3828	2.9627	6.0044
		r	0.9854	0.9998	0.9997	0.9989
		MRE	0.8440	0.9994	0.9980	0.9939
Training	Outer stress23	$R^{2}$	0.9711	0.9997	0.9995	0.9979
		RMSE(MPa)	31.9624	2.2926	2.8705	5.8425
		r	0.9854	0.9998	0.9997	0.9989
		MRE	0.8440	0.9994	0.9980	0.9940
Training	Outer stress24	$R^{2}$	0.9713	0.9997	0.9995	0.9981
		RMSE(MPa)	31.9216	2.1632	2.7335	5.6057
		r	0.9855	0.9999	0.9998	0.9990
		MRE	0.8439	0.9994	0.9979	0.9938
Training	Outer stress25	$R^{2}$	0.9715	0.9998	0.9996	0.9982
		RMSE(MPa)	31.8719	1.9911	2.5583	5.3340
		r	0.9856	0.9999	0.9998	0.9991
		MRE	0.8438	0.9993	0.9980	0.9939

**Table A2:** Detailed prediction accuracy of four RF models in the testing datasets.

Dataset	Output	Parameters	RF	ACO-RF	GA-RF	GWO-RF
Testing	Inner stress1	$R^{2}$	0.8333	0.9011	0.8957	0.8495
		RMSE(MPa)	30.0074	20.6258	21.2353	25.4910
		r	0.9128	0.9493	0.9464	0.9217
		MRE	0.7923	0.9478	0.9307	0.9394
Testing	Inner stress2	$R^{2}$	0.8836	0.9389	0.9394	0.9113
		RMSE(MPa)	43.3498	26.3288	26.3931	31.8236
		r	0.9400	0.9690	0.9692	0.9546
		MRE	0.8032	0.9362	0.9197	0.9422
Testing	Inner stress3	$R^{2}$	0.9215	0.9678	0.9678	0.9540
		RMSE(MPa)	48.5299	24.3547	24.4654	29.1799
		r	0.9599	0.9838	0.9838	0.9767
		MRE	0.8367	0.9849	0.9668	0.9788
Testing	Inner stress4	$R^{2}$	0.9298	0.9751	0.9748	0.9658
		RMSE(MPa)	52.1086	24.0963	24.3433	28.2712
		r	0.9643	0.9875	0.9873	0.9827
		MRE	0.8340	0.9912	0.9697	0.9843
Testing	Inner stress5	$R^{2}$	0.9347	0.9814	0.9804	0.9747
		RMSE(MPa)	53.3165	21.9952	22.6736	25.6728
		r	0.9668	0.9907	0.9902	0.9873
		MRE	0.8300	0.9909	0.9672	0.9844
Testing	Inner stress6	$R^{2}$	0.9436	0.9874	0.9861	0.9826
		RMSE(MPa)	47.7223	17.1131	18.0405	20.1384
		r	0.9714	0.9937	0.9930	0.9913
		MRE	0.8320	0.9937	0.9681	0.9883
Testing	Inner stress7	$R^{2}$	0.9488	0.9906	0.9894	0.9867
		RMSE(MPa)	44.5806	14.2618	15.2075	17.0074
		r	0.9740	0.9953	0.9947	0.9933
		MRE	0.8330	0.9960	0.9676	0.9912
Testing	Inner stress8	$R^{2}$	0.9517	0.9924	0.9910	0.9880
		RMSE(MPa)	41.1745	12.1184	13.1978	15.1982
		r	0.9755	0.9962	0.9955	0.9940
		MRE	0.8344	0.9963	0.9654	0.9925
Testing	Inner stress9	$R^{2}$	0.9556	0.9946	0.9935	0.9912
		RMSE(MPa)	38.9538	9.8531	10.7713	12.5363
		r	0.9775	0.9973	0.9968	0.9956
		MRE	0.8363	0.9980	0.9647	0.9941
Testing	Inner stress10	$R^{2}$	0.9577	0.9956	0.9948	0.9927
		RMSE(MPa)	37.2435	8.5667	9.3696	11.0404
		r	0.9786	0.9978	0.9974	0.9964
		MRE	0.8378	0.9986	0.9637	0.9947
Testing	Inner stress11	$R^{2}$	0.9598	0.9966	0.9961	0.9946
		RMSE(MPa)	36.2926	7.3965	7.9909	9.3966
		r	0.9797	0.9983	0.9980	0.9973
		MRE	0.8390	0.9992	0.9634	0.9948
Testing	Inner stress12	$R^{2}$	0.9611	0.9972	0.9969	0.9956
		RMSE(MPa)	35.5853	6.6028	7.0655	8.3446
		r	0.9803	0.9986	0.9984	0.9978
		MRE	0.8398	0.9992	0.9629	0.9946
Testing	Inner stress13	$R^{2}$	0.9619	0.9977	0.9974	0.9963
		RMSE(MPa)	35.2565	6.0549	6.4021	7.5898
		r	0.9808	0.9988	0.9987	0.9982
		MRE	0.8401	0.9989	0.9625	0.9940
Testing	Inner stress14	$R^{2}$	0.9626	0.9980	0.9978	0.9968
		RMSE(MPa)	34.9817	5.6354	5.9180	7.0750
		r	0.9811	0.9990	0.9989	0.9984
		MRE	0.8402	0.9986	0.9621	0.9936
Testing	Inner stress15	$R^{2}$	0.9630	0.9981	0.9980	0.9970
		RMSE(MPa)	34.8404	5.3932	5.6272	6.7780
		r	0.9813	0.9991	0.9990	0.9985
		MRE	0.8400	0.9982	0.9617	0.9931
Testing	Inner stress16	$R^{2}$	0.9632	0.9982	0.9981	0.9971
		RMSE(MPa)	34.7398	5.2758	5.4786	6.6374
		r	0.9814	0.9991	0.9990	0.9986
		MRE	0.8399	0.9979	0.9615	0.9929
Testing	Inner stress17	$R^{2}$	0.9634	0.9982	0.9981	0.9972
		RMSE(MPa)	34.6737	5.2354	5.4115	6.5781
		r	0.9815	0.9991	0.9991	0.9986
		MRE	0.8397	0.9975	0.9613	0.9926
Testing	Inner stress18	$R^{2}$	0.9634	0.9982	0.9981	0.9972
		RMSE(MPa)	34.6493	5.2507	5.4101	6.5832
		r	0.9815	0.9991	0.9991	0.9986
		MRE	0.8396	0.9974	0.9613	0.9926
Testing	Inner stress19	$R^{2}$	0.9635	0.9982	0.9981	0.9972
		RMSE(MPa)	34.6228	5.2743	5.4211	6.5955
		r	0.9816	0.9991	0.9990	0.9986
		MRE	0.8395	0.9972	0.9613	0.9926
Training	Inner stress20	$R^{2}$	0.9634	0.9982	0.9981	0.9972
		RMSE(MPa)	34.6354	5.3047	5.4504	6.6210
		r	0.9816	0.9991	0.9990	0.9986
		MRE	0.8394	0.9972	0.9613	0.9926
Training	Inner stress21	$R^{2}$	0.9634	0.9982	0.9981	0.9972
		RMSE(MPa)	34.6333	5.3122	5.4636	6.6256
		r	0.9815	0.9991	0.9990	0.9986
		MRE	0.8393	0.9971	0.9613	0.9926
Training	Inner stress22	$R^{2}$	0.9634	0.9982	0.9981	0.9972
		RMSE(MPa)	34.6692	5.3070	5.4776	6.6240
		r	0.9815	0.9991	0.9990	0.9986
		MRE	0.8393	0.9971	0.9613	0.9926
Training	Inner stress23	$R^{2}$	0.9633	0.9982	0.9981	0.9972
		RMSE(MPa)	34.6816	5.2596	5.4559	6.5837
		r	0.9815	0.9991	0.9990	0.9986
		MRE	0.8394	0.9971	0.9614	0.9928
Training	Inner stress24	$R^{2}$	0.9633	0.9982	0.9981	0.9972
		RMSE(MPa)	34.7238	5.2050	5.4311	6.5364
		r	0.9815	0.9991	0.9990	0.9986
		MRE	0.8394	0.9972	0.9615	0.9925
Training	Inner stress25	$R^{2}$	0.9633	0.9983	0.9981	0.9973
		RMSE(MPa)	34.7004	5.1365	5.3591	6.4637
		r	0.9815	0.9991	0.9991	0.9986
		MRE	0.8396	0.9976	0.9619	0.9929
Training	Outer stress1	$R^{2}$	0.8626	0.9310	0.9377	0.9216
		RMSE(MPa)	228.5337	139.2439	134.0324	149.4037
		r	0.9288	0.9649	0.9683	0.9600
		MRE	0.7988	0.9959	0.9637	0.9871
Training	Outer stress2	$R^{2}$	0.8772	0.9524	0.9541	0.9441
		RMSE(MPa)	125.7301	66.8862	66.7746	73.0712
		r	0.9366	0.9759	0.9768	0.9717
		MRE	0.8086	0.9966	0.9677	0.9873
Training	Outer stress3	$R^{2}$	0.8754	0.9614	0.9590	0.9467
		RMSE(MPa)	84.0521	40.0646	42.1035	47.4426
		r	0.9356	0.9805	0.9793	0.9730
		MRE	0.8170	0.9984	0.9691	0.9913
Training	Outer stress4	$R^{2}$	0.8695	0.9606	0.9548	0.9363
		RMSE(MPa)	56.2323	26.6666	29.0192	33.9125
		r	0.9324	0.9801	0.9771	0.9676
		MRE	0.8314	0.9956	0.9647	0.9960
Training	Outer stress5	$R^{2}$	0.8951	0.9657	0.9627	0.9504
		RMSE(MPa)	43.6513	20.9249	22.0404	25.1714
		r	0.9461	0.9827	0.9812	0.9749
		MRE	0.8424	1.0033	0.9608	1.0019
Training	Outer stress6	$R^{2}$	0.9147	0.9685	0.9675	0.9589
		RMSE(MPa)	38.1363	18.8254	19.2575	21.5230
		r	0.9564	0.9841	0.9836	0.9793
		MRE	0.8472	0.9915	0.9544	1.0037
Training	Outer stress7	$R^{2}$	0.9297	0.9756	0.9756	0.9693
		RMSE(MPa)	38.2698	17.6189	17.7062	19.7856
		r	0.9642	0.9877	0.9877	0.9845
		MRE	0.8463	0.9964	0.9550	0.9949
Training	Outer stress8	$R^{2}$	0.9398	0.9825	0.9822	0.9775
		RMSE(MPa)	37.7454	15.3956	15.6351	17.5188
		r	0.9694	0.9912	0.9911	0.9887
		MRE	0.8445	0.9965	0.9559	0.9942
Training	Outer stress9	$R^{2}$	0.9454	0.9866	0.9861	0.9822
		RMSE(MPa)	38.2383	14.0717	14.4067	16.2491
		r	0.9723	0.9933	0.9930	0.9910
		MRE	0.8425	0.9902	0.9566	0.9899
Training	Outer stress10	$R^{2}$	0.9513	0.9906	0.9899	0.9872
		RMSE(MPa)	37.3270	11.9179	12.4167	13.9183
		r	0.9754	0.9953	0.9949	0.9936
		MRE	0.8414	0.9952	0.9587	0.9908
Training	Outer stress11	$R^{2}$	0.9547	0.9927	0.9920	0.9900
		RMSE(MPa)	36.9708	10.6362	11.1501	12.4684
		r	0.9771	0.9963	0.9960	0.9950
		MRE	0.8406	0.9965	0.9599	0.9909
Training	Outer stress12	$R^{2}$	0.9576	0.9944	0.9938	0.9923
		RMSE(MPa)	36.2671	9.2766	9.8119	10.9439
		r	0.9786	0.9972	0.9969	0.9961
		MRE	0.8403	0.9975	0.9609	0.9913
Training	Outer stress13	$R^{2}$	0.9595	0.9956	0.9951	0.9938
		RMSE(MPa)	35.8826	8.3239	8.8035	9.8354
		r	0.9796	0.9978	0.9975	0.9969
		MRE	0.8401	0.9978	0.9612	0.9913
Training	Outer stress14	$R^{2}$	0.9609	0.9964	0.9960	0.9949
		RMSE(MPa)	35.5053	7.4623	7.9044	8.8854
		r	0.9803	0.9982	0.9980	0.9975
		MRE	0.8402	0.9983	0.9617	0.9917
Training	Outer stress15	$R^{2}$	0.9618	0.9970	0.9967	0.9957
		RMSE(MPa)	35.2867	6.8290	7.2204	8.1810
		r	0.9807	0.9985	0.9983	0.9978
		MRE	0.8402	0.9984	0.9619	0.9918
Training	Outer stress16	$R^{2}$	0.9624	0.9974	0.9972	0.9962
		RMSE(MPa)	35.1262	6.3195	6.6799	7.6498
		r	0.9810	0.9987	0.9986	0.9981
		MRE	0.8404	0.9986	0.9621	0.9921
Training	Outer stress17	$R^{2}$	0.9627	0.9977	0.9975	0.9966
		RMSE(MPa)	35.0213	5.9656	6.2997	7.2877
		r	0.9812	0.9989	0.9987	0.9983
		MRE	0.8404	0.9985	0.9620	0.9920
Training	Outer stress18	$R^{2}$	0.9630	0.9979	0.9977	0.9968
		RMSE(MPa)	34.9611	5.7150	6.0330	7.0471
		r	0.9813	0.9989	0.9988	0.9984
		MRE	0.8405	0.9985	0.9621	0.9922
Training	Outer stress19	$R^{2}$	0.9631	0.9980	0.9978	0.9969
		RMSE(MPa)	34.9034	5.5474	5.8534	6.8920
		r	0.9814	0.9990	0.9989	0.9985
		MRE	0.8405	0.9985	0.9620	0.9921
Training	Outer stress20	$R^{2}$	0.9632	0.9981	0.9979	0.9970
		RMSE(MPa)	34.8780	5.4398	5.7353	6.7971
		r	0.9814	0.9990	0.9989	0.9985
		MRE	0.8405	0.9985	0.9620	0.9921
Training	Outer stress21	$R^{2}$	0.9632	0.9981	0.9980	0.9971
		RMSE(MPa)	34.8325	5.3598	5.6459	6.7275
		r	0.9814	0.9991	0.9990	0.9985
		MRE	0.8405	0.9984	0.9620	0.9921
Training	Outer stress22	$R^{2}$	0.9633	0.9982	0.9980	0.9971
		RMSE(MPa)	34.8105	5.3006	5.5712	6.6697
		r	0.9815	0.9991	0.9990	0.9986
		MRE	0.8405	0.9984	0.9619	0.9921
Training	Outer stress23	$R^{2}$	0.9634	0.9982	0.9981	0.9972
		RMSE(MPa)	34.7570	5.2292	5.4848	6.5962
		r	0.9815	0.9991	0.9990	0.9986
		MRE	0.8404	0.9982	0.9618	0.9920
Training	Outer stress24	$R^{2}$	0.9634	0.9983	0.9981	0.9972
		RMSE(MPa)	34.7314	5.1838	5.4132	6.5320
		r	0.9815	0.9991	0.9991	0.9986
		MRE	0.8402	0.9980	0.9615	0.9916
Training	Outer stress25	$R^{2}$	0.9634	0.9983	0.9982	0.9973
		RMSE(MPa)	34.6875	5.1162	5.3313	6.4471
		r	0.9815	0.9992	0.9991	0.9987
		MRE	0.8400	0.9974	0.9612	0.9912
